# Comparison of developmental milestone attainment in early treated HIV-infected infants versus HIV-unexposed infants: a prospective cohort study

**DOI:** 10.1186/s12887-017-0776-1

**Published:** 2017-01-17

**Authors:** Sarah Benki-Nugent, Dalton Wamalwa, Agnes Langat, Kenneth Tapia, Judith Adhiambo, Daisy Chebet, Helen Moraa Okinyi, Grace John-Stewart

**Affiliations:** 1 Department of Global Health, University of Washington, Box 359909, 325 9th Ave., Seattle, WA 98104 USA; 2 Department of Paediatrics and Child Health, University of Nairobi, Nairobi, Kenya

**Keywords:** Antiretroviral therapy, HIV, Infant, Sub-Saharan Africa, Neurodevelopment, Early antiretroviral therapy

## Abstract

**Background:**

Infant HIV infection is associated with delayed milestone attainment. The extent to which effective antiretroviral therapy (ART) prevents these delays is not well defined.

**Methods:**

Ages at attainment of milestones were compared between HIV-infected (initiated ART by age <5 months), and HIV-unexposed uninfected (HUU) infants. Kaplan Meier analyses were used to estimate and compare (log-rank tests) ages at milestones between groups. Adjusted analyses were performed using Cox proportional hazards models.

**Results:**

Seventy-three HIV-infected on ART (median enrollment age 3.7 months) and 92 HUU infants (median enrollment age 1.6 months) were followed prospectively. HIV-infected infants on ART had delays in developmental milestone attainment compared to HUU: median age at attainment of sitting with support, sitting unsupported, walking with support, walking unsupported, monosyllabic speech and throwing toys were each delayed (all *p*-values <0.0005). Compared with HUU, the subset of HIV-infected infants with both virologic suppression and immune recovery at 6 months had delays for speech (delay: 2.0 months; *P =* 0.0002) and trend to later walking unsupported. Among HIV-infected infants with poor 6-month post-ART responses (lacking viral suppression and immune recovery) there were greater delays versus HUU for: walking unsupported (delay: 4.0 months; *P =* 0.0001) and speech (delay: 5.0 months; *P* < 0.0001).

**Conclusions:**

HIV infected infants with viral suppression on ART had better recovery of developmental milestones than those without suppression, however, deficits persisted compared to uninfected infants. Earlier ART may be required for optimized cognitive outcomes in perinatally HIV-infected infants.

**Trial registration:**

NCT00428116; January 22, 2007.

**Electronic supplementary material:**

The online version of this article (doi:10.1186/s12887-017-0776-1) contains supplementary material, which is available to authorized users.

## Background

Ninety percent of HIV-infected infants worldwide are born in sub-Saharan Africa, where opportunities for early HIV diagnosis are frequently missed [1–4]. Infant HIV diagnosis often does not occur until after onset of symptomatic HIV [5–7]. HIV-infected infants have a particularly high early viral burden [8] and rapid disease progression [9], and the window of opportunity to minimize the HIV reservoir in the brain is likely short. Early pre-ART disease progression may be associated with neuronal damage that is not salvageable. Conversely, effective ART may provide benefit, if initiated very early in infancy. In cohorts of infants with no or limited access to early ART, 30–36% and 26–36% had mental and motor delays, respectively [10–12].

There are few data on neurodevelopmental outcomes of early ART-treated infants in Africa. In ART-treated HIV-infected children in the US, early (prior to age 5 years) plasma viral suppression [13] was associated with higher school-age IQ scores, supporting the hypothesis that effective ART in infancy may improve neurocognition; however, in this study children with better virologic responses to ART still had scores well below national norms. In older US cohorts, children with no history of symptomatic HIV disease had similar neurocognitive outcomes compared with HIV-exposed uninfected peers [14–17], but these studies potentially included an elite subset of surviving HIV-infected children.

Recent data from the South African CHER study suggests that early ART is broadly beneficial in preserving neurocognitive function. In this randomized trial, asymptomatic infants without immunosuppression who received ART by 6–12 weeks of age had similar mental, social and locomotor scores as HIV-uninfected infants at 11 months of age [18]. In the same study, infants who received deferred ART (until symptomatic or meeting CD4 treatment criteria at the time) had lower locomotor scores, suggesting early HIV disease progression compromised outcomes.

For HIV-infected infants born in settings in which symptomatic HIV prior to ART is the norm, it is unknown whether an effective response to ART during early infancy can lead to comparable neurodevelopmental outcomes as HIV-uninfected peers. We previously reported that greater CD4% gain over 6 months on ART was associated with earlier age at walking and speech in early treated Kenyan infants, a majority of whom who presented with symptomatic HIV disease at ART [19]. Here, we present data comparing this cohort of HIV-infected infants to HUU infants with a similar age range. We hypothesized that these infants would have persistent differences in milestone attainment compared with HUU infants, even if they had early and effective responses to ART.

## Methods

### Study population

HIV-infected infants participated in a randomized clinical trial (Optimizing Pediatric HIV-1 Therapy 03 (OPH03) with a 2-year pre-randomization period, in which infants attended monthly study visits, with data collection, prior to randomization (NCT00428116) [20]. As described previously, infants were identified at either maternal child health clinics or hospital wards in Nairobi. Enrollment eligibility criteria were HIV DNA PCR positive with confirmation, age <5 months, and no previous receipt of antiretroviral therapy, with the exception of prophylaxis for prevention of mother to child transmission (PMTCT). Following enrollment and adherence counseling, ART was initiated, generally within 2 weeks. Infants with no prior exposure to nevirapine (NVP) for PMTCT received a first-line regimen consisting of NVP plus 2 nucleoside reverse transcriptase inhibitors (generally zidovudine and lamivudine), and infants with prior NVP exposure received a first-line regimen consisting of this backbone, plus ritonavir-boosted lopinavir (LPV/r). Nearly all infants had received trimethoprim-sulfamethoxazole (cotrimoxazole) prophylaxis prior to or at enrollment. HIV-infected infants were enrolled from 2007 to 2009.

From 2011 to 2013, HIV-unexposed uninfected (HUU) infants were identified following routine HIV-testing offered to pregnant mothers at the Mathare North MCH Clinic in Nairobi. HIV seronegative mothers were recruited for infant participation in a 2-year cohort study involving similar study visit schedules as in the OPH03 study. Enrollment eligibility criteria were 1) age 0–4.5 months, 2) biological mother and infant with HIV-negative status, and 3) mother planned to reside within Nairobi for at least 2 years. Mother and infant HIV status were verified using an HIV rapid antibody test at enrollment. Maternal HIV-negative status was confirmed at 12 and 24 months during follow-up.

Following standardized questionnaires on sociodemographic and medical history, infants received a physical examination at enrollment and a blood sample was collected for CD4 testing and for HIV-infected infants, virologic testing. During follow-up, infants attended monthly visits at which a physical exam was administered with blood collection at 1-month and 3-monthly thereafter for HIV-positive infants, and 6-monthly for HUU infants.

### Developmental milestones

Developmental milestones were assessed at enrollment and at monthly visits for the duration of follow-up. Milestones were adapted from the Denver Developmental Screening Test [21]. The same team of clinical officers performed study visits and assessed milestones. Caregivers self-reported the age at which the milestone had been achieved at monthly visits. Analyses presented here utilized the self-reported age wherever possible, and an estimated age (using the date of study visit at which the milestone was first noted by the clinician) was used in cases in which the self-reported age was missing. Sensitivity analyses including only the clinician-reported age gave similar results. Milestones included full neck control (defined as whether the infants could support his or her own head) (neck control), sitting with support (defined as whether the child could sit with a straight back on the caregiver’s lap or on the floor between the caregiver’s legs), sitting unsupported (defined as whether the child could sit on a flat surface or on the caregiver’s lap without needing support), walking with support (defined as whether the child could walk well with the help of someone holding one or both hands or when supporting self using furniture or a wall), walking unsupported (defined as whether the child could take a few steps without needing support), monosyllabic speech (defined as whether the infant was able to say one-syllable words or sounds, referring to a specific person or object) (speech), throwing toys (defined as whether the child could throw a toy such as a ball while playing), naming objects (defined as whether the caregiver reported that the child could point to and name common household items such as cups or plates). This analysis was focused primarily on milestones expected to occur within a few months of the 6-month on-ART visit, namely walking unsupported and speech.

### Laboratory testing

In Nairobi, flow cytometry was performed to obtain enrollment and 6-monthly CD4 counts and percentages. Whole blood specimens were centrifuged using a Ficoll density gradient to separate plasma, which was cryopreserved at −70 ° C. Plasma HIV RNA levels were ascertained using the Gen-Probe HIV Viral Load Assay (San Diego, CA) in Seattle, WA [22]. Blood hemoglobin levels were assessed in Nairobi.

### Statistical analysis

This study was a secondary analysis using data collected for infants participating in a randomized clinical trial (all infants with available milestone data were included) and a comparison group of infants enrolled (*N* = 100) during a later timeframe. Z-scores for weight-for-age (WAZ), height-for-age (HAZ), weight-for-height (WHZ) and head circumference-for-age (HCZ) were calculated using the 2006 WHO reference population [23]. Baseline characteristics were compared between HUU and HIV-infected infants using t-tests for continuous variables and chi-squared tests or Fisher’s exact tests, as appropriate, for dichotomous variables. Baseline cofactors considered were infant sex, birth weight, hemoglobin level, and z-scores for growth parameters, and caregiver age, marital status, years of education, number of rooms in the house, and household monthly rent.

Median (50^th^ percentile) and the interquartile range (IQR) (25^th^ and 75^th^ percentiles) for ages at attainment of milestones were calculated using Kaplan-Meier survival methods, with birth as the time origin. Thirty-one (42.5%) HIV-infected infants and 13 (17.3%) HUU infants already had achieved neck control by the time of enrollment, and thus the proportion of infants with neck control by age 5.0 months (the maximum age at enrollment for these 44 infants) was compared. Age at attainment of milestones was compared between groups using log-rank tests for all other milestones. Using this approach, infants who never achieved a given milestone contributed to the analysis until they were censored.

Multivariate analyses employed Cox proportional hazards regression. In addition to baseline cofactors listed above, we evaluated WAZ during ART (6-months post-start), average monthly change in WAZ, and a dichotomous indicator for WAZ < −2 during ART for inclusion in multivariate models. Cofactors were assessed for association with age at walking unsupported or speech within either HIV-infected or HUU infants; those with significance *P* < 0.05 were evaluated in multivariate modeling. Multivariate models including caregiver education were also evaluated a priori.

Primary analyses focused on the walking unsupported and speech milestones. For these analyses, infants with effective response to ART (defined using virologic, immune, and growth markers) were compared with HUU infants as a referent. HIV-infected infants with effective 6-month viral, immune, and growth responses were defined as having plasma HIV RNA <1000 HIV-RNA copies/ml, CD4% ≥25%, and WAZ ≥ −2 at 9 months of age (approximately 6 months post initiation of ART), respectively. For primary multivariate analysis, the threshold for considering statistical significance utilized a conservative Bonferroni adjustment for the 6 multiple comparisons (three groups defined by 6-month virologic, immune, and growth responses for walking unsupported and speech). Therefore, *P*-values <0.008 were considered statistically significant. Results are also shown for the comparison of the ineffective ART groups to HUU.

In exploratory analyses, infants with combined 6-month immune and viral responses to ART were compared with HUU infants. For each of these stratified analyses, ages of attainment of walking unsupported and speech for HIV-infected infants with poor immune, virologic, or growth responses, or combined poor immune and virologic responses were also compared with that for HUU infants. In addition, exploratory analyses compared age at speech in infants who received LPV/r-based ART compared with HUU infants.

Statistical analyses were performed using Stata SE, version 11.2, College Station, TX.

## Results

### Enrollment characteristics of HIV-infected and HUU infants

Ninety-nine HIV-infected infants were enrolled, of whom 26 died, were lost-to-follow-up or withdrew before initiating ART and providing milestone data, leaving 73 for these analyses [20] (see Additional file 1: Figure S1). Among 100 enrolled HUU infants, seven were lost-to-follow-up or withdrew prior to providing milestone data, and one was diagnosed with cerebral palsy and was excluded from analysis. At enrollment, HIV-infected infants were older than HUU infants (medians, 3.7 vs 1.6 months; *P* < 0.0001) (Table 1). HIV-infected infants had lower birth weight (3.1 vs 3.2 kg) *P* = 0.02), and baseline WAZ (medians, −2.0 vs −0.2; *P* < 0.0001), HAZ (medians, −1.9, vs −1.4; *P* = 0.04), WHZ (medians, −0.6, vs 1.4; *P* < 0.0001), and HCZ (medians, −0.5 vs 0.4; *P* = 0.0002) compared with HUU infants. HIV-infected infants had a median CD4 percentage of 18%, 30 (41.1%) were diagnosed with WHO Stage 3 or 4 disease, and 38 (52.1%) had been hospitalized prior to enrollment. Sixty-two of seventy-three (84.9%) infants had a CD4 percentage below 25%, were diagnosed with WHO Stage 3 or 4 HIV disease, or both, before starting ART.

**Table 1:** Summary of baseline characteristics for infants included in analysis

	HIV-infected		HUU		
	N^a^	Median (IQR) or N (%)	N^a^	Median (IQR) or N (%)	P
Infant characteristics					
Age at enrollment (months)	73	3.7 (3.1, 4.0)	92	1.6 (0.9, 2.1)	<0.0001
Male	73	35 (48.0)	92	53 (57.6)	0.2
Birth weight (kg)	68	3.1 (2.7, 3.4)	92	3.2 (3.0, 3.5)	0.02
Received PMTCT	68	39 (57.4)	-	-	-
Hemoglobin g/dL	73	9.7 (9, 10.9)	79	11.0 (10.2, 12.7)	<0.0001
Ever breastfed	64	56 (87.2)	89	89 (100)	0.001^b^
Infant clinical, immunologic, virologic, and growth status					
Ever hospitalized	73	38 (52.1)	92	0 (0)	<0.001^b^
WHO stage 3 or 4	73	30 (41.1)	-	-	-
Plasma HIV RNA (log_10_ copies/mL)	69	6.5 (6.0, 7.0)	-	-	-
CD4%	73	18 (14, 24)	80	42 (35, 47)	<0.0001
CD4 count (cells/mL)	73	1,311 (801, 1,760)	80	2,515 (2,011, 3,244)	<0.0001
WAZ	73	−2.0 (−3.3, −0.9)	92	−0.2 (−1.0, 0.3)	<0.0001
HAZ	73	−1.9 (−3.0, −0.8)	92	−1.4 (−2.4, −0.4)	0.04
WHZ	73	−0.6 (−1.6, 0.6)	92	1.4 (0.2, 2.3)	<0.0001
HCZ	73	−0.5 (−1.3, 0.5)	92	0.4 (−0.5, 1.1)	0.0002
Primary caregiver characteristics					
Biological mother	73	71 (97.3)	92	92 (100)	-
Age (years)	72	26 (22, 30)	89	24 (22, 27)	0.1
Married	73	57 (78.1)	91	80 (87.9)	0.09
Education (years)	66	9 (8, 11)	90	9 (8, 12)	0.3
One-room house	73	56 (76.7)	91	75 (82.4)	0.4
Household monthly rent (KES)	69	1,500 (1,000, 2,500)	88	2,500 (2,000, 3,500)	<0.0001
Maternal CD4 count (cells/mm^3^)	70	370 (246, 478)	-	-	

*HUU* HIV-unexposed uninfected, *IQR* interquartile range, *PMTCT* prevention of mother-to-child transmission, *WHO* World Health Organization, *WAZ* weight-for-age z-score, *HAZ* height-for-age z-score, *WHZ* weight-for-height z-score, *HCZ* head circumference-for-age z-score, *KES* Kenyan Shillings

^a^Includes infants surviving, or remaining enrolled in the study long enough to define attainment of neck control

^b^Fisher’s exact test

The majority of HIV-infected infants (97.3%) and (per eligibility criteria) all HUU infants were cared for by their biological mother. The majority of caregivers in each group of infants were married (78.1% vs 87.9%; *P* = 0.09). Both groups had similar education levels (medians, 9 vs 9 years; *P* = 0.3), but HIV-infected had lower monthly rent levels (medians, 1,500 vs 2,500 KES; *P* < 0.0001). The majority of infants in each group lived in a one-room house (76.7% vs 82.4%; *P* = 0.4).

### Delayed milestones in HIV-infected versus HUU infants

We previously reported that prior history of hospitalization, a WHO Stage 3 or 4 diagnosis, lower WAZ, HAZ, and WHZ and lower maternal CD4 count were associated with later age at milestone attainment in HIV-infected infants [19]. In the present analysis, HUU infants living in a house with >1 room had earlier age at attainment of milestones (speech, medians, 11 (IQR, 9, 12) vs 12 (IQR, 11, 13) months; *P* = 0.02; throwing toys, medians, 14 (IQR, 13, 15) vs 15 (IQR 14, 16) months; *P* = 0.02). Infants living in households with higher rent (>1500 KES/month) also had earlier age at throwing toys (medians, 14 (IQR, 14, 16); vs 17 (IQR, 15, 18) months; *P* = 0.02).

We hypothesized that HIV-infected infants would have later milestone attainment compared with HUU infants. In Kaplan-Meier analyses, 82.8% (95% CI, 72.9, 90.6) of HIV-infected and 96.6% (95% CI, 91.2, 99.1) of HUU infants had neck control by 5 months of age. HIV-infected infants also had significantly later age at sitting unsupported (medians, 7 vs 6 months; *P* < 0.0001), walking unsupported (medians, 15 vs 13 months; *P* < 0.0001) and speech (medians, 15 months vs 12; *P* = 0.0001), compared with HUU infants (Table 2). Age at attainment of other milestones, including sitting with support, walking with support, and throwing toys were also significantly later compared with HUU. Median age at naming objects was marginally later for HIV-infected and HUU infants, though this did not reach statistical significance (22 versus 19 months; (*P* = 0.07).

**Table 2:** Comparison of age at attainment of milestones for HIV-infected infants vs HIV - unexposed uninfected infants using Kaplan Meier survival analyses

	HIV-infected		HUU		
	Events	Median (IQR) Months	Events	Median (IQR) Months	P^a^
Milestone					
Sitting with support	63	5 (5, 6)	91	4 (4, 5)	<0.0001
Sitting unsupported	61	7 (6, 7)	88	6 (5, 6)	<0.0001
Walking with support	56	9 (10, 12)	77	9 (8, 10)	0.0002
Walking unsupported	54	15 (13, 18)	69	13 (11, 14)	<0.0001
Monosyllabic speech	53	15 (13, 17)	72	12 (10, 13)	0.0001
Throwing toys	55	17 (15, 19)	60	15 (14, 16)	<0.0001
Naming objects	52	22 (19, 24)	45	19 (18, 23)	0.07

*HUU* HIV-unexposed uninfected, *IQR* interquartile range

^a^Log - rank tests

### Growth, immune and virologic responses during ART were associated with partial improvement for age at attainment of milestones

**Fig. 1:**
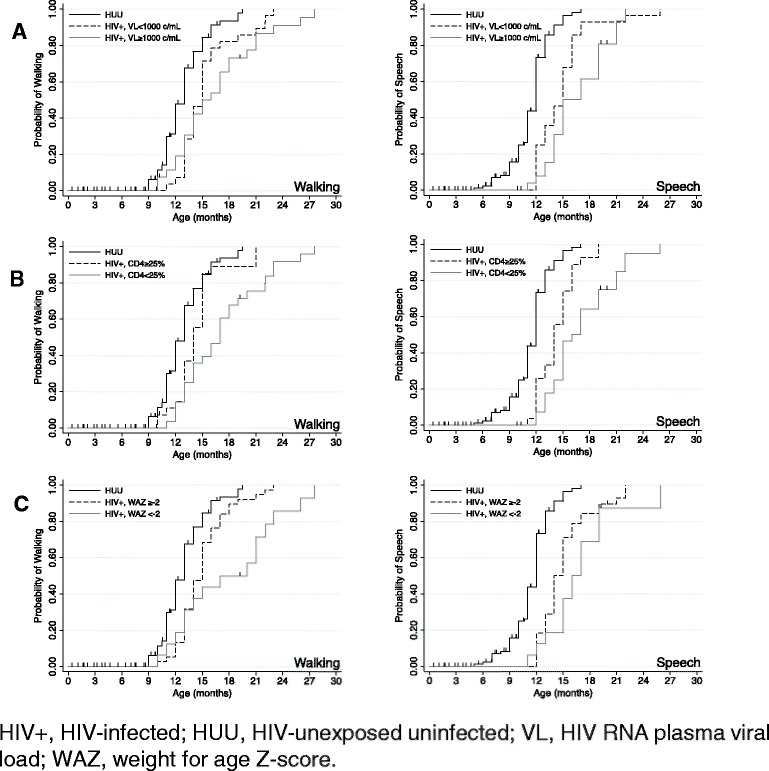
Kaplan Meier survival curves comparing probability of walking unsupported and speech between HIV-infected and HIV-unexposed uninfected (HUU) infants (stratified by **a**, virologic response at 6 months post-ART (approximately 9 months of age); **b**, immune response; **c**, growth response)

At 6 months post-ART start, 28/54 (51.8%) of infants had viral suppression (plasma HIV RNA <1000 copies/ml), 27 (50.0%) had immune reconstitution (CD4% ≥25), and 38 (70.4%) infants had achieved a WAZ ≥ −2. As would be expected based on prior studies [13, 24], HIV-infected infants with poor 6-month viral responses to ART (plasma HIV RNA ≥1000 copies/ml) had significantly later speech and walking unsupported milestones than HUU infants (all *P*-values ≤0.0001; Table 3 and Fig. 1). These differences remained significant in analyses adjusted for 6 month post-ART growth (WAZ < −2) and household rent, (all *P*-values <0.005). Results were similar for infants with poor immune response to ART and underweight (WAZ < −2) at 6-months following start of ART.

**Table 3:** Kaplan-Meier (unadjusted) and Cox proportional regression (adjusted) analyses for age at attainment of milestones for HIV-infected infants stratified by ART responses and ART regimen and compared with HIV-unexposed uninfected infants

	Unadjusted ^b^			Adjusted ^c^	
	N^a^	Median (IQR) Months	P	HR (95% CI)	P
Walking unsupported					
HUU-reference	69	13 (11, 14)			
HIV+, 6-mo VL <1000 c/mL	28	15 (13, 16)	0.0009	0.58 (0.36, 0.92)	0.02*
HIV+, 6-mo VL ≥1000 c/mL	25	16 (13, 20)	0.0001	0.43 (0.24, 0.76)	0.004
HIV+, 6-mo CD4% ≥25%	27	14 (13, 15)	0.02	0.73 (0.46, 1.16)	0.2*
HIV+, 6-mo CD4% <25%	27	17 (13, 20)	<0.0001	0.35 (0.19, 0.61)	<0.001
HIV+, 6-mo WAZ ≥ −2	38	15 (13, 16)	0.001	0.59 (0.39, 0.90)	0.014*
HIV+, 6-mo WAZ < −2	15	17 (13, 22)	<0.0001	0.26 (0.13, 0.53)	<0.001
Monosyllabic speech					
HUU-reference	72	12 (10, 13)		-	-
HIV+, 6-mo VL <1000 c/mL	28	15 (12, 16)	<0.0001	0.28 (0.16, 0.46)	<0.001*
HIV+, 6-mo VL ≥1000 c/mL	24	15 (14, 19)	<0.0001	0.20 (0.11, 0.36)	<0.001
HIV+, 6-mo CD4% ≥25%	27	14 (12, 16)	<0.0001	0.33 (0.21, 0.55)	<0.001*
HIV+, 6-mo CD4% <25%	26	16 (14, 19)	<0.0001	0.14 (0.08, 0.27)	<0.001
HIV+, 6-mo WAZ ≥ −2	37	14 (13, 16)	<0.0001	0.26 (0.16, 0.42)	<0.001*
HIV+, 6-mo WAZ < −2	15	16 (15, 19)	<0.0001	0.15 (0.07, 0.29)	<0.001

*HUU* HIV-unexposed uninfected, *HIV+ * HIV-infected, *IQR* interquartile range, *CI* confidence interval, *VL* plasma viral load (HIV RNA), *WAZ* weight - for - age Z-score

^a^N, number of events

^b^ Comparison of each group of HIV-infected infants vs HIV-unexposed uninfected infants (HUU) using log - rank tests

^c^ Cox regression analyses adjusted for monthly household rent. For comparisons of effective viral load and CD4%, adjustment was also made for WAZ < −2 at 9 months of age (approximately 6 months after initiating ART)

*Using Bonferroni to account for 6 multiple comparisons, *P*-values <0.008 were considered statistically significant

We hypothesized that infants with effective responses to ART (defined by their virologic, immune or growth responses) would have significant developmental differences compared with HUU infants. HIV-infected infants with viral suppression had delayed walking unsupported and speech compared to HUU (walking median delay, 2 months; *P* = 0.0009; speech median delay, 3 months; *P* <0.0001).

In Cox proportional hazards models adjusted for 6 month post-ART growth status (WAZ < −2) and household rent, infants with viral suppression had significantly later speech (adjusted hazard ratio (aHR), 0.28, 95% confidence interval (CI), 0.16, 0.46; *P* < 0.001) and a trend to later walking unsupported (aHR, 0.58, 95% CI, 0.36, 0.92; *P =* 0.02, above the Bonferroni-corrected alpha of 0.008). Infants with 6-month immune reconstitution had delayed speech compared to HUU (aHR, 0.33, 95% CI, 0.21, 0.55; *P* < 0.001). Infants with sustained WAZ > −2 at 6 months also had later speech (*P* <0.001). Pre-ART WAZ, 6-month change in WAZ, birth weight, living in a one-room house, and caregiver education were not included in final models due to collinearity. In sensitivity analyses, models adjusted for each of these variables gave similar results.

Eighteen of fifty-four (33.3%) infants had combined viral suppression and immune reconstitution at 6-months post-ART. In exploratory analyses, these infants had similar age at walking unsupported (aHR, 0.73, 95% CI 0.43, 1.26; *P* = 0.3), but had delayed speech compared with HUU infants (unadjusted median delay, 2 months; *P =* 0.0002; aHR, 0.40, 95% CI, 0.23, 0.69; *P =* 0.001). Infants with both poor viral suppression and poor immune reconstitution had even more pronounced delays (walking: median difference delay, 4 months; *P =* 0.0001; speech delay, 5 months, *P* < 0.0001) compared with HUU infants. These differences remained in adjusted models (all *p*-values ≤0.001).

HIV-infected infants in this study received LPV/r-based ART if they had prior exposure to NVP for PMTCT, and we previously reported that infants receiving LPV/r-based ART had significantly earlier speech compared with those receiving NVP-ART [19]. In exploratory analyses, infants receiving LPV/r-based ART still had significantly later speech than HUU infants (aHR, 0.37, 95% CI, 0.21, 0.65; *P* = 0.001).

## Discussion

Here we show that early ART-treated HIV-infected infants had measurable neurodevelopmental differences compared with HUU infants in the first 2 years of life. These results are consistent with recent findings from the CHER trial in South Africa, in which infants randomized to deferred ART had lower locomotor scores at 11 months of age compared with HIV-uninfected infants [18]. In addition, a second South African study found differences in language and motor scores among HIV-infected infants who had initiated ART at a mean age of approximately 5 months and were followed for six months, compared with HIV-exposed uninfected infants [25]. In contrast to CHER, which did not find deficits in other domains, we found persistent delays in speech attainment among early-ART treated infants compared to HUU, even in the subgroups with evidence of good ART response with viral suppression, immune recovery and growth. Compared with HUU infants, HIV-infected infants with both viral suppression and immune reconstitution had significantly later speech. These results suggest that among HIV-infected infants who often had HIV symptoms prior to initiation of ART, neurodevelopmental delays may not be completely resolved despite effective ART responses.

Data from previous studies suggest a narrow window of time during infancy in which neurocognitive deficits due to HIV may be reversed by ART. In the PREDICT study, asymptomatic children over 1 year of age had no improvement in neurocognitive outcomes during nearly 3 years of follow-up on ART [26]. A retrospective analysis including children ages 2–17 years and with CDC Class 1 or 2 disease demonstrated only modest improvement in vocabulary scores following initiation of PI-based therapy [27]. In the same study, children with viral suppression at one-year following combination ART did not demonstrate better neurocognitive functioning [27]. Thus, among children over 1 year of age, neurocognitive effects of HIV may not be reversed by ART in the same way as among infants receiving ART in the first months of life.

Previous studies have found deficits in language in HIV-infected children [25, 28–30]. We previously reported that use of LPV/r-based ART was associated with earlier age for speech in this cohort. Here, we further show that in spite of benefits for earlier speech in HIV-infected children receiving LPV/r-based ART, their speech was still delayed compared to HUU children.

Delays in milestone attainment in spite of systemic viral suppression or immune reconstitution may reflect early viral infiltration to the central nervous system or poor clearance from the CNS following ART. In South African infants, white matter abnormalities were apparent on magnetic resonance imaging scans in 50% of 44 early treated infants who had suspected HIV-related neurological disease, many of whom had viral suppression prior to imaging [31]. Similarly, a case study identified 4 children with cognitive decline in spite of viral suppression [32]. Neurocognitive impairment in spite of effective suppressive ART has also been described in adults [33]. Alternatively or in parallel, later milestone attainment in HIV-infected infants may reflect poor early infant health, which may have delayed physical and social play activities important for speech and walking attainment.

Stunting and micronutrient deficiencies are key cofactors for poor neurodevelopment in the general population [34], and are common in perinatally HIV-infected children [35–37]. Poor growth in HIV-infected infants may be further compounded by the metabolic cost of HIV and other co-pathogens and chronic immune activation in the periphery. We previously reported that among HIV-infected infants, those with lower nutritional status both prior to ART and during ART had later walking attainment [19]. Here we show that infants with adequate growth (WAZ > −2) during ART had a trend for later walking unsupported and significantly later speech compared with HUU infants. We found that living in a one-room house was associated with delayed speech attainment in HUU infants, underscoring the interplay between economic status and neurodevelopment. Malnutrition and poverty combined with HIV may have synergistic detrimental effects on long-term neurocognitive outcomes. It is likely that a combined approach incorporating social interventions, nutritional supplements, and ART are necessary to fully optimize developmental outcomes in HIV-infected children receiving ART.

HUU infants were recruited from a clinic with a majority of clientele living within an informal settlement of Nairobi. Most HUU infants in our study lived in one-room housing, as did HIV-infected infants. Both groups had substantial stunting (37.0 and 46.6%, respectively), compared with a Kenyan national prevalence of 10% stunting among infants aged less than 6 months (2014 Kenyan Demographic Health Survey) [38]. Compared with the 2006 WHO reference population based in Ghana, India, Norway, Oman and the US, HUU infants in our study had slightly later walking unsupported (12.0 vs 13.0 months) [39]. Growth and developmental differences between this HUU group and national and international norms may be partly attributable to socioeconomic differences.

Our study did not include an HIV-exposed uninfected comparison group, and it is possible that infants with better ART responses may have similar outcomes compared with HIV-exposed uninfected children. HIV-exposure is associated with higher morbidity, malnutrition, and socioeconomic difficulties, such as HIV stigma, orphan-hood or severe parental illness, and poverty, all of which could contribute to delayed milestones [24, 40]. In cohorts from Uganda and Thailand, HIV-exposed uninfected children had deficits in receptive language and global cognitive functioning, respectively [30, 41].

We used parental self-report of a small number of developmental milestones. A limitation of this study is lack of detailed assessments of infant developmental functioning for motor, social and language domains. In addition, the HIV-infected and HUU cohorts were enrolled during different timeframes (4–6 years apart). The two cohorts differed in their catchment sites: HIV-infected infants were either identified in hospital or maternal child health clinics (MCH) in Nairobi; whereas HUU infants were recruited from a single MCH clinic. There was overlap in the study clinical officers who evaluated infants in each cohort, and we are not aware of any programmatic differences that would have led to different environmental stimulation between the cohorts. We cannot rule out the possibility that subtle differences in data collection may have occurred between cohorts.

Strengths of this study included the intensive follow-up (monthly), which allowed high precision for age at attainment of milestones, growth, immune and virologic data. HIV-infected infants in this study received empiric ART [42], but were often immunocompromised, and thus these results are generalizable to many HIV-infected infants in sub-Saharan Africa who do not receive their HIV diagnosis until onset of HIV symptoms have occurred.

## Conclusions

In summary, we demonstrate partial restoration of neurodevelopmental outcomes in HIV-infected infants with immune reconstitution and viral suppression by 6 months post-ART, compared to uninfected peers. Growth status during ART and immune and viral response to ART were each independent cofactors for age at milestone attainment. These data suggest that effective early ART is an important intervention for ensuring optimal neurodevelopment in HIV-infected infants, but that additional strategies, including nutritional and social support, are likely needed.

## Additional file

Additional file 1: Flow chart of selected milestones for HIV-infected (A) and HIV-unexposed (B) infant cohorts. (DOCX 116 kb)

## Abbreviations

ART: Antiretroviral therapy
c: Copies
HAZ: Height-for-age z-score
HCZ: Head circumference-for-age z-score
HIV+: HIV-infected
HUU: HIV-unexposed uninfected
IQR: Interquartile range
KES: Kenyan Shillings
LPV/r: Ritonavir-boosted lopinavir
NVP: Nevirapine
PMTCT: Prevention of mother-to-child transmission
VL: Plasma viral load (HIV RNA)
WAZ: Weight-for-age z-score
WHO: World Health Organization
WHZ: Weight-for-height z-score
