# Supplementary material for: Comparison of developmental milestone attainment in early treated HIV-infected infants versus HIV-unexposed infants: a prospective cohort study
Source: BMC Pediatr. 2017 Jan 17;17:24. doi: 10.1186/s12887-017-0776-1 (PMC5240280; doi:10.1186/s12887-017-0776-1)
Supplement: Additional file 1: — Flow chart of selected milestones for HIV-infected (A) and HIV-unexposed (B) infant cohorts. (DOCX 116 kb) [file 12887_2017_776_MOESM1_ESM.docx]

**Additional File 1.** Flow chart of selected milestones for HIV-infected (A) and HIV-unexposed uninfected (B) infant cohorts

**A B**

7 lost/withdrawn

1 with cerebral palsy

18 died

8 lost/withdrawn

100 HIV-unexposed uninfected infants

99 HIV-infected infants

8 died

4 lost/withdrawn

4 lost/withdrawn

92 with age at neck control

73 with age at neck control

3 died

4 lost/withdrawn

1 died

15 lost/ withdrawn

88 with age at sitting unsupported

61 provided age at sitting unsupported

3 lost/withdrawn^^^

69 with age at walking unsupported

72 with age at speech

1 lost*

54 with at age at walking unsupported

53 with age at speech

*Did not provide age at speech.

^Did not provide age at walking.
